# Viscosity Characteristics of Cationic Polyacrylamide Aqueous Solutions

**DOI:** 10.3390/polym18030331

**Published:** 2026-01-26

**Authors:** Mamdouh T. Ghannam, Mohamed Y. E. Selim, Ahmed Thaher, Nejood Ahmad, Reem Almarzooqi, Afnan Khalil

**Affiliations:** 1 Department of Chemical and Petroleum Engineering, College of Engineering, United Arab Emirates University, Al-Ain P.O. Box 15551, United Arab Emirates; 2 Mechanical and Aerospace Engineering Department, College of Engineering, United Arab Emirates University, Al-Ain P.O. Box 15551, United Arab Emirates

**Keywords:** cationic polyacrylamide, viscosity, shear rate, shear stress, sodium chloride, calcium chloride

## Abstract

This investigation evaluates the viscosity and flow performance of cationic polyacrylamide (CPAA) solutions by assessing the effect of CPAA concentrations, shear rate, temperature, and electrolyte salt types. The study aims to characterize the flow behavior of CPAA solutions for different industrial utilizations under some challenging conditions of high salinity of two different electrolytes and high-temperature environments. In addition, the study addresses the critical shear rate thresholds at which the transition from shear-thinning to shear-thickening occurs. An Anton Paar rotational rheometer was employed to evaluate the flow behavior of cationic polyacrylamide solutions over the range of 20–80 °C at 20 °C intervals. Polymer samples were prepared from CPAA powder in a concentration range of 500–5000 ppm. To determine the electrolyte effects, NaCl and CaCl_2_ were incorporated into the polymer solutions with a concentration range of 0–10 Wt.%. This study revealed that shear stress is vastly sensitive to CPAA concentration at shear rates less than 200 s^−1^, whereas this sensitivity reduces at higher shear rates where the resulting profiles converge. Moreover, a considerable decrease in shear stress was reported with temperature as a result of the thermal influence on the molecular interaction forces. Rheological analysis of the CPAA solutions shows they exhibit strong non-Newtonian shear-thinning behaviors with viscosity decreasing significantly as the shear rate approaches 200 s^−1^. On the contrary, a transition to a shear-thickening profile is observed at a shear rate above this limit of 200 s^−1^. The results show that the dynamic viscosity of the CPAA solutions rises significantly as the concentration increases from 500 to 5000 ppm. At a shear rate of 10 s^−1^, the dynamic viscosity increased from 2.4 to 33.8 mPa·s as the CPAA concentration increased from 500 to 5000 ppm (exactly 2.4, 11.8, 16.6, and 33.8 mPa.s for 500, 1500, 2500, and 5000 ppm, respectively). Additionally, increasing the temperature from 20 to 80 °C exerts a strong negative influence on dynamic viscosity. Specifically, for the 5000 ppm concentration at a shear rate of 10 s^−1^, the dynamic viscosity decreased from 33.8 to 18.3 mPa.s as the temperatures rose from 20 to 80 °C (recorded as 33.8, 27.9, and 18.3 mPa.s at 20, 40, and 80 °C, respectively). Furthermore, the introduction of different electrolytes, such as NaCl and CaCl_2_, significantly reduces the viscosity flow profiles.

## 1. Introduction

Cationic polyacrylamide copolymer (CPAA) is a widely utilized polymer in numerous industrial applications such as in food development, agronomy, waste treatment processing, water treatment flocculant, sand and coal washing, wastewater treatment, and sludge dewatering applications. Cationic polyacrylamide copolymer is prepared from the copolymerization of acrylamide and cationic monomers which have a positive charge. Cationic polyacrylamide copolymer is a water-soluble polymer that can be widely utilized in papermaking and oil extraction thickener.

One of the effective applications for the polyacrylamide is in the oilfield arena and, in particular, for enhanced oil recovery, in which around of 50–60% of the original oil remains unproduced by the traditional techniques of primary and secondary production methods. Enhanced oil recovery (EOR) techniques have been developed in the past few decades to recover more of the trapped oil in the oil reservoir [1,2,3,4]. According to many of the published articles, the majority of the produced crude oil in the coming times will heavily rely on EOR methods [5,6,7,8,9]. Different methods are available for EOR, such as CO_2_ and steam injections, however the polymer injection choice is more commonly employed to enhance oil extraction and to stretch the lifespan of the oilfield production [3,4,10,11,12]. Some of the main objectives of the polymer injections within EOR are to displace some of the trapped oil or to drive the chemical fluids within the oil formation [13,14]. Among the endorsed synthetic polymers commonly employed in EOR are polyacrylamides [15]. Anionic polyacrylamide polymers are recommended for sandstone wells, as the repulsive forces generated between the negative charges of the anionic polyacrylamide polymers and those on the rock surfaces will lower the effect of the adsorption and the viscosity losses as well [16]. On the other hand, regarding carbonate wells, in which almost half of the world’s oil is trapped, anionic polyacrylamide will be challenging to utilize, due to the chemical interactions generated between the negative charges of the anionic polymers and the positive charges of the well walls. These generated attractive interactions will lead to a significant increase of both polymer viscosity losses and polymer adsorption on the rock surfaces [17]. Therefore, for such carbonate wells, it is recommended to consider the utilization of cationic polyacrylamide polymers owing to the repulsive forces that will be generated between the positive charges of the injected polymers and the rock surfaces [18]. Many rheological studies have been carried out on polyacrylamide aqueous solutions. Some of these representative investigations include a study of the flow aspects of these solutions [19], the assessment of mechanical deficiency at ultrahigh deformation rates during hydraulic fracture [20], and evaluations of polyacrylamide solutions for turbulent pipe flow [21].

Cationic polyacrylamide CPAA is a highly effective polymer with diverse applications; however, it possesses several limitations concerning its physical preparation, ecological safety, and chemical stability. Among these limitations are the challenging dissolution process; the solid form of CPAA dissolves slowly, typically requiring approximately 60 min of gradual addition into stirred, hot double-distilled water to avoid the formation of insoluble fish-eyes. As it is a synthetic polymer, CPAA exhibits poor biodegradability and is classified as hazardous to marine ecosystems. CPAA is highly susceptible to mechanical, thermal, and chemical degradation, which can lead to a loss of viscosity and functional efficiency. Its deterioration is further accelerated when exposed to high temperatures or humidity. CPAA is generally more expensive than its anionic or non-ionic counterparts, with prices increasing significantly as the molecular weight rises.

As highlighted earlier, cationic polyacrylamide polymers can be considered in many practical industrial operations in food, agriculture, waste treatment, water flocculant, and enhanced oil recovery. Consequently, for these applications and others, it would be necessary to recognize the rheological descriptions of the cationic polyacrylamide polymer solutions to be able to obtain better a understanding of the flow details of these polymer solutions. The rheology field allows assessing the solution flow characteristics, through the effect of the applied shear stress or strain, to obtain rheogram behaviors in terms of shear stress (τ) or dynamic viscosity (η) versus shear rate ($\dot{\gamma }$) profiles [22,23,24].

A full understanding of these rheological properties is essential to designate the flow details of the CPAA polymer solutions for various processes such as preparation, storing, heating, cooling, and EOR flooding injection. Therefore, the objectives of the current study are to systematically determine the steady-state flow profiles of CPAA solutions over a wide range of shear rates, assess the impact of four different temperatures on dynamic viscosity profiles, and evaluate the comparative effects of two electrolyte salts on the rheological properties of CPAA. Furthermore, this study determines the critical shear rate thresholds at which the transition from shear-thinning to shear-thickening arises.

## 2. Materials and Methods

### 2.1. Materials

Chinafloc cationic polyacrylamide was delivered from Innoveda International General Trading LLC (Dubai, United Arab Emirates). It is a water-soluble off-white granular powder with a bulk density of 0.75 g/cm^3^ and a size range of almost 850 μm (98% of the whole sample). It is often used as a flocculant in water treatment, and also in food processing, agriculture, and wastewater treatment as well. The pH of 1 Wt.% is approximately 6.5 at 25 °C. Cationic polyacrylamide is a high-molecular-weight copolymer of 15–16 million g/mol. Chinafloc cationic polyacrylamide is characterized by a linear chain structure formed by the copolymerization of non-ionic backbone acrylamide monomers providing the structural length with positively charged cationic monomers. The general formula for the polyacrylamide backbone is (–CH_2_CHCONH_2_–)_n_. In its cationic form, some of these units are replaced by cationic monomers, resulting in a positively charged polyelectrolyte. The charge density represents the proportion of cationic monomers relative to total monomers in the polymer chain. For the Chinafloc cationic polyacrylamide supplied by Innoveda International General Trading LLC (Dubai, United Arab Emirates), the polymer chain charge is not a single fixed value but rather a customizable range to suit specific needs. Based on the technical specifications delivered for the Chinafloc, the polymer chain charge is in the medium range of 20–40%.

Four polymer solution samples with concentrations of 500, 1500, 2500, and 5000 ppm were prepared to examine a wide range of CPAA concentrations. This selection of a 500–5000 ppm concentration range for evaluating the flow behavior of cationic polyacrylamide is based on CPAA fundamental physics and rheological requirements. For example, in the field of EOR, a concentration range of 500–3000 ppm is often utilized to achieve the necessary mobility control and viscosity level. In another application, for example, wastewater treatments, the stock solution is usually prepared within the range of 1000–5000 ppm for complete dissolution before final dilution to ensure the necessary flocculation efficiency.

### 2.2. Methods

Experimental samples of the cationic polyacrylamide were prepared by mixing a specific amount of CPAA within 250 cm^3^ of hot double distilled water around 45 °C to enable the formation of total dissolved polymer aqueous solutions. A sufficient time of almost one hour was allowed to form a homogeneous solution without any external mixing to avoid unwanted consequences, such as polymer network degradation.

The rheometer of MCR92 (from Anton Paar, Graz, Austria) was utilized for the current experimental investigation of the cationic polyacrylamide solution flow behavior, which allows obtaining the required measurement outcomes in the rotation mode of the used rheometer. This mode allows the MCR92 unit to implement the rheological measurement results within the controlled-rate mode functions. The MCR92 rheometer consists of coaxial cylinders of Bob and Cup configuration, in which the diameters of the Bob and Cup units are 26.6 and 28.9 mm, respectively, with a length of 40 mm. A water circulating system was attached to the MCR92 rheometer to control the applied temperature. At the beginning of each rheological test, a standard oil was employed to calibrate the MCR92 unit to confirm that the used rheometer will have the appropriate conditions to implement the planned and required tests. Once the calibration step was completed in an acceptable way, the proper amount of the prepared cationic polyacrylamide polymer sample was transferred into the assigned measuring cup up to the specified level. This stage was tracked by inserting the Bob device fully inside the sample of the polymer solution. To guarantee the consistency and reliability of the experimental measurements of the current study, at the onset of an experimental run, a designated set of measurements was repeated three times using the same settings. The acceptance level of such a stage was about ±2–3%.

## 3. Results and Discussion

The main emphasis of the present investigation is to address the flow characteristics of the cationic polyacrylamide aqueous solutions in terms of shear stress (Pa) and dynamic viscosity (mPa.s) against the assigned shear rate (s^−1^) for different polymer concentrations over the range of 500–5000 ppm polymer. This experimental investigation involved the effect of shear rate over the range of 0–500 s^−1^ and a temperature range of 20–80 °C in 20 °C increments, and two different types of electrolyte salts of NaCl and CaCl_2_ over the concentration range of 0–10 Wt.% to detect their effects on the flow profiles of the examined polymer solutions.

### 3.1. Flow Profiles of Cationic Polyacrylamide Solutions

**Figure 1:**
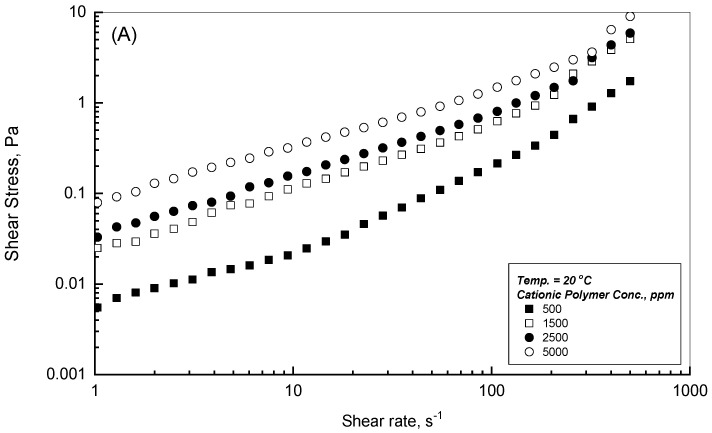

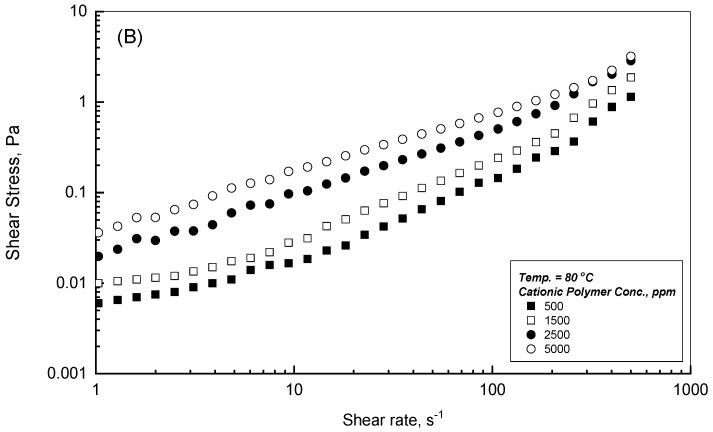
Flow profiles of the different cationic polymer concentrations at low and high temperatures (**A**) 20 °C; (**B**) 80 °C.

The rheogram behavior in terms of shear stress (Pa) versus shear rate (s^−1^) was utilized to observe the flow profiles of the CPAA aqueous solutions over the range of polymer concentrations of 500–5000 ppm. These results of shear stresses and shear rates cover three cycles of shear rate over 1–500 s^−1^. Figure 1A,B demonstrate the rheogram behaviors for the low and high assigned temperatures of 20 and 80 °C. As can be seen from these illustrations in Figure 1A,B, the resulting shear stresses gradually increase with the increasing assigned shear rate and the concentration of the CPAA. On the logarithmic scales, Figure 1A,B show that the effect of the polymer concentrations is more pronounced at a shear rate of less than 200 s^−1^; beyond this level, the polymer concentrations of 1500, 2500, and the highest of 5000 ppm are approaching each others’ profiles regardless of the polymer concentration, indicating that the shear effect is more dominant to dictate their viscosity level.

**Figure 2:**
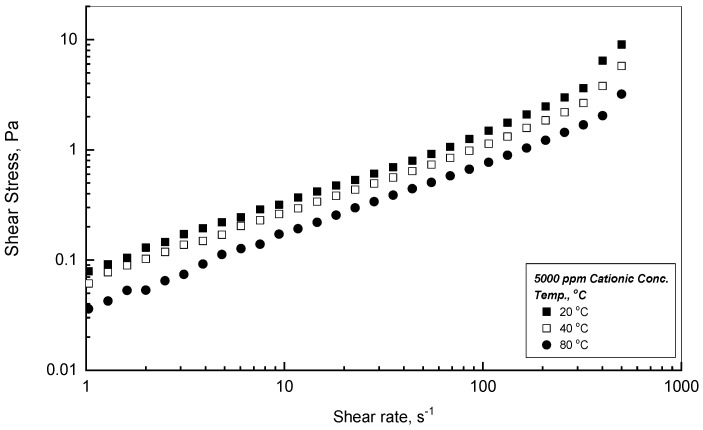
Thermal effect on the flow profiles of the 5000 ppm polymer concentration.

The general observation of the earlier illustrations at 20 and 80 °C shows that the thermal effect exhibits a significant impact on the shear stress–shear rate profiles, which may be detected evidently by examining Figure 2 over the temperature range of 20–80 °C. Figure 2 displays the rheogram behaviors for the CPAA of 5000 ppm solution at three values of different temperatures. Figure 2 shows that the tested polymer solution of CPAA decreases steadily and noticeably with the applied temperature increase from 20 to 80 °C, which can be ascribed to the adverse effects of the thermal influence on the molecular interaction and cohesion forces of the tested polymer solutions.

Simulation analysis is a necessary tool to acquire further knowledge of the CPAA flow performance for the experimental measurements illustrated in Figure 1 and Figure 2. This process of examination is valuable to attain a mathematical formula to use for flow contour prediction and formation. The SigmaPlot program was used for the purpose of inspecting the experimental data illustrated in Figure 1 and Figure 2 to determine the suitable mathematical formulas that best fit those data. According to the displayed data in Figure 1 and Figure 2, the two mathematical formulas of Equations (1) and (2) can be considered to simulate the flow behaviors of Figure 1 and Figure 2. These formulas are the Power law (Ostwald–de Waele model) of Equation (1) and the second model of the Herschel–Bulkley of Equation (2), which associate the shear stress–shear rate relationships.

τ = m ˙γ^n^ (1)

τ = τ_o_ + m ˙γ^n^ (2)

where ˙γ denotes the applied shear rate in s^−1^, τ is the corresponding shear stress in Pa, m is the consistency index in Pa.s^n^, n is the flow behavior index, and τ_o_ is the apparent yield stress in Pa. According to the illustrations of Figure 1 and Figure 2, the Power law model fitted some of those measurements, whereas the outcomes of the SigmaPlot software v15.1 showed that the Herschel–Bulkley model simulated the whole set of the experimental measurements of Figure 1 and Figure 2 impressively, providing regression coefficients (i.e., R^2^) near the value of 1, as reported in Table 1 and Table 2.

Table 1 reports the simulation results for the apparent yield stress τ_o_, the consistency index m, and the flow behavior index n at two temperature values of 20 and 80 °C, respectively. As can be concluded from these results, τ_o_ steadily increases with the concentration of CPAA from 0.0133 to 0.2208 Pa at 20 °C and from 0.0125 to 0.0607 Pa at 80 °C within the concentration of 500 to 5000 ppm. The existence of the reported yield stress of τ_o_ can be attributed to the three-dimensional network formation in the absence of shear environments, which rises with higher polymer addition. In the presence of assigned shear on the examined polymer solution, the three-dimensional network will decline, and the polymer solution displays shear-thinning behavior. Table 2 reveals a substantial reduction for the apparent yield stress and the flow behavior index values with temperature from 20 to 80 °C due to the thermal effect on the CPAA solution characteristics.

### 3.2. Viscosity Profiles

**Figure 3:**
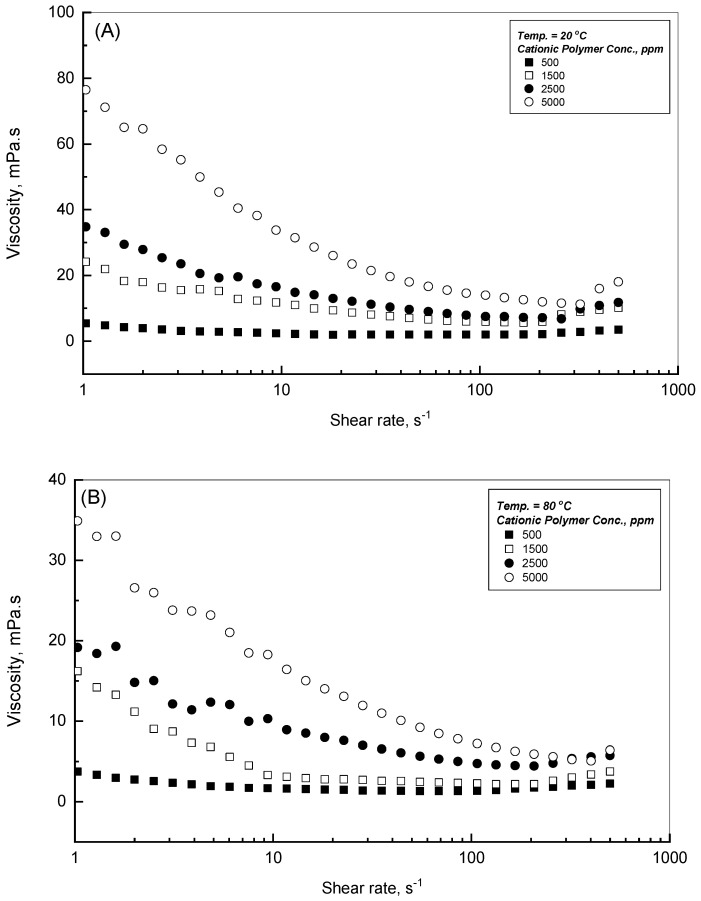
Viscosity profiles of the cationic polymer at (**A**) 20 and (**B**) 80 °C.

One of the measurement capabilities accompanying the rheometer of MCR 92 is its ability to convey the measurement outcomes in terms of dynamic viscosity values against the assigned shear rates for the tested polymer samples. The viscosity flow profiles in mPa.s versus the assigned shear rate in s^−1^ on semi-logarithmic scales are illustrated in Figure 3A,B for different CPAA concentrations of 500–5000 ppm. This investigation examines a wide range of shear rates with the range of 1–500 s^−1^ in which it covers three cycles on the logarithmic scale. Two different levels of low and high temperatures, 20 and 80 °C, were studied and are displayed in Figure 3A,B, respectively. These illustrations for all CPAA polymer solution concentrations show non-Newtonian strong shear-thinning flow profiles with the assigned shear rate in which the viscosity values decrease significantly with shear rate until the level of 200 s^−1^. This is a representative shear-thinning behavior of the polymer solutions, in which the reported dynamic viscosity values deteriorate with the applied shear rate. The reported viscosity value drop versus the effect of shear rate can be attributed to the unlooping and coordination of the CPAA polymer chains that will occur during the effect of the shear rate.

On the other side, Figure 3 shows a slight rise in the dynamic viscosity values that can be observed with shear rate beyond the reported level of 200 s^−1^, depicting a contrary behavior of shear-thickening behavior. It has been observed in other investigations that shear-thickening will take place at a critical shear rate of ˙γ_c_, which was reported for some polymer solutions of high molecular weight, such as in the results stated by, for example, Ait-Kadi et al., Rivero et al., Jiang et al., and Gürgen et al. [25,26,27,28]. This reversed shear-thickening behavior can be described as owing to the flow-induced reforming assembly that will occur over time, and depends upon the applied shear rate. Regarding the effect of the polymer concentration, the measured dynamic viscosity values increase strongly with CPAA concentration from 500 to the 5000 ppm, as can be concluded from the observation of Figure 3, which shows that the higher CPAA polymer growth of concentration leads to much higher dynamic viscosity values. For instance, the dynamic viscosity improved from 2.4 to 33.8 mPa.s with CPAA concentration increasing from 500 to 5000 ppm at a shear rate of 10 s^−1^ (specifically, the reported values of 2.4, 11.8, 16.6, and 33.8 mPa.s for 500, 1500, 2500, and 5000 ppm, respectively).

The shear-thinning transition to shear-thickening is a rheological complexity status that arises for semi-dilute or concentrated polyacrylamide solutions. This phenomenon is triggered mainly by changes in polymer chain arrangement and their resulting interactions with the assigned shear rate. At a shear rate lower than a critical value of ˙γ_c_, the polymer solutions exhibit shear-thinning behavior with declining viscosity, owing to the arrangement of polymer chains in the flow path. These arrangements reduce the hydrodynamic volume and interchain entanglements. As the shear rate surpasses the critical rate of ˙γ_c_, the CPAA solution changes to a shear-thickening behavior. This transition is due to the shear-induced structure (i.e., coil-stretch and inter-chain interactions) and hydrodynamic clustering (i.e., the temporary formation of large-scale clusters), which rely on the polymer molecular weight, concentration, and temperature. The critical shear rate of ˙γ_c_ is the shear rate that initiates the shear-thickening behavior, which is the experimentally observed value that can vary based on polymer molecular weight, polymer concentration, and temperature [25,26,27,28].

### 3.3. Effect of Temperature on Cationic Polyacrylamide Flow Behavior

**Figure 4:**
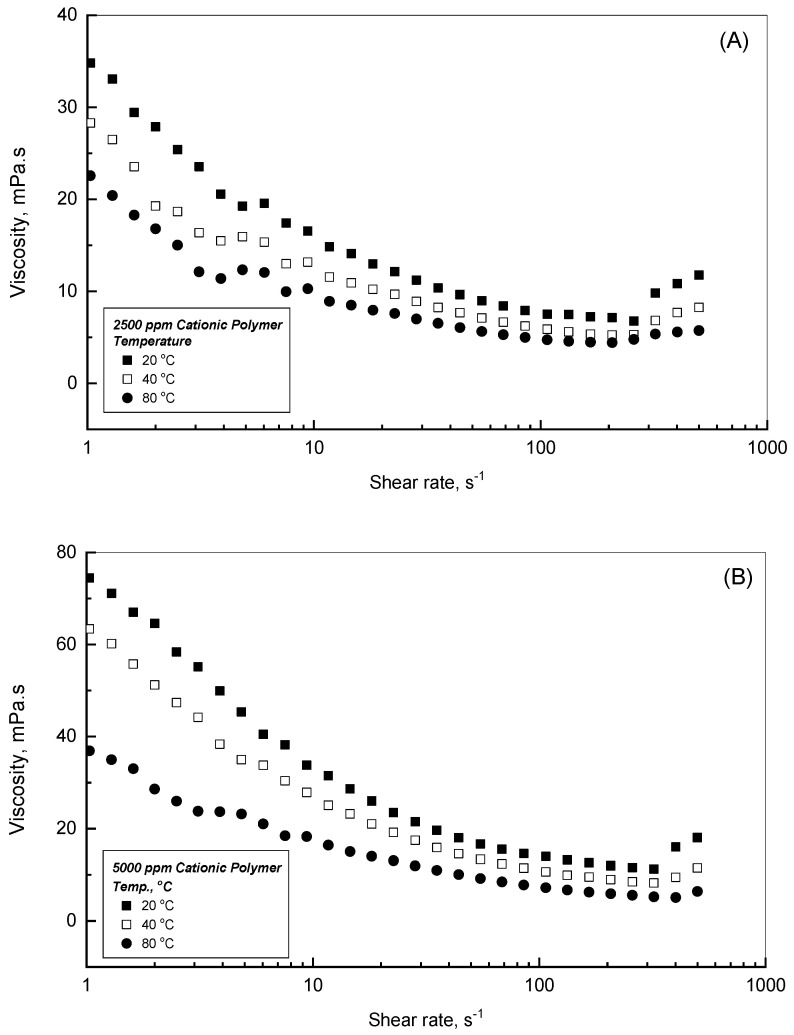
Thermal effect on the viscosity profiles for the (**A**) 2500 and (**B**) 5000 ppm cationic polymer.

It is a useful practice to observe the thermal effect on the flow behavior of the CPAA solutions. Therefore, the dynamic viscosity profiles versus the applied shear rates for two different concentrations of 2500 and 5000 ppm aqueous solutions are displayed in Figure 4A and Figure 4B, respectively. This investigation examines a wide temperature range, covering 20–80 °C in 20 °C increments, to perceive and scrutinize the resulting behaviors. These illustrations show a strong thermal effect on the viscosity flow profiles. As the temperature increases from 20 to 80 °C, Figure 4A,B show that the CPAA viscosity measurements deteriorate significantly. For example, in the case of 2500 ppm solution and a shear rate of 1 s^−1^, the dynamic viscosity decreases from 34.8 to 28.3 and 22.6 mPa.s for the temperatures of 20, 40, and 80 °C, respectively, while at a shear rate of 10 s^−1^, the viscosity will be 16.6, 13.2, and 10.3 mPa.s for the temperatures of 20, 40, and 80 °C, respectively. However, for the 5000 ppm polymer solution and shear rate of 1 s^−1^, Figure 4B shows that the dynamic viscosity declines from 74.5 to 63.4 and 36.9 mPa.s for the temperatures of 20, 40, and 80 °C, respectively. However, at a 10 s^−1^ shear rate, the dynamic viscosity will drop to 33.8, 27.9, and 18.3 mPa.s for temperatures of 20, 40, and 80 °C, respectively. Owing to higher temperatures, the molecular motion enhances within the polymer solution, which depresses the interaction period with the nearest entities. Hence, the generated intermolecular forces of the present molecules will be adversely affected. However, the solution viscosity will be improved due to the electrostatic repulsions of the positive charges on the molecular chains, which increases polymer hydrolysis at higher temperatures. It is worthwhile to note that the viscosity improvement that resulted from polymer hydrolysis is less dominant than the viscosity drops that resulted from the adverse impact of temperature on the intermolecular forces.

### 3.4. Effect of NaCl Addition

**Figure 5:**
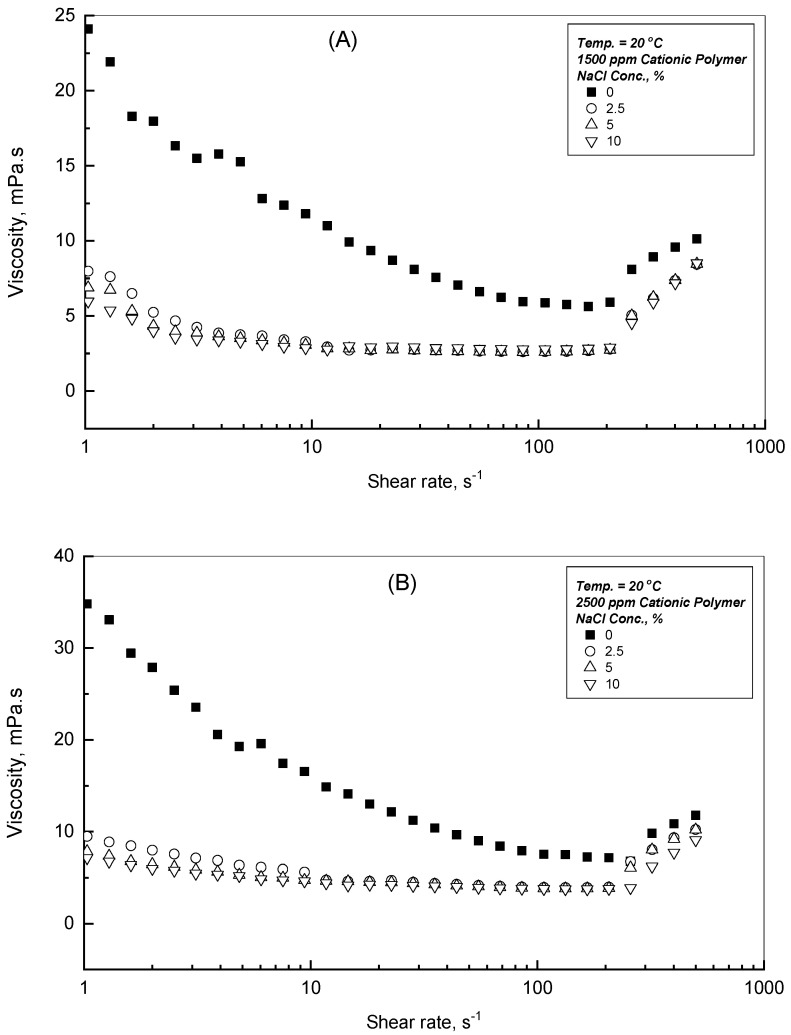

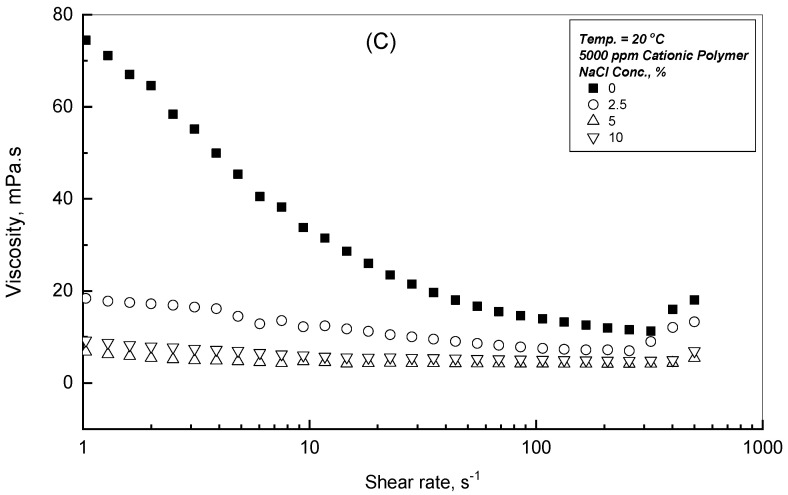
Effect of NaCl on the viscosity profiles for various CPAA concentrations at 20 °C, (**A**) 1500 ppm Cationic Polymer; (**B**) 2500 ppm Cationic Polymer; (**C**) 5000 ppm Cationic Polymer.

The earlier discussion mentioned that the viscosity of the CPAA solution enhances with the polymer addition, while it decreases with both the shear rate and applied temperature. For the current study, it is advantageous to assess the following behavior in terms of the dynamic viscosity of the CPAA solutions in the presence of NaCl concentration within the range of 0–10 Wt.% to perceive the effect of salinity concentration on the CPAA flow behaviors. To examine the effect of NaCl concentration, Figure 5A–C display the viscosity flow profiles of the CPAA concentrations for 1500, 2500, and 5000 ppm at 20 °C as typical examples. As can be seen from the provided illustrations, the presence of the added NaCl diminishes the viscosity flow profiles significantly for all the CPAA concentrations, and even more noticeably for the higher polymer concentration of 5000 ppm, as shown in Figure 5C. To assess and evaluate this observation in more detail, Table 3, Table 4 and Table 5 report the viscosity reduction% for each CPAA concentration at selected shear rates of 0.1, 1.0, and 10.0 s^−1^, respectively. The results reported in Table 3, Table 4 and Table 5 show that the viscosity reduction% increases with NaCl concentration and shear rate up to the value of 10 s^−1^.

The dynamic viscosity of CPAA in distilled water, in salt-free media, is significantly high, and it increases even more with greater addition of CPAA, because the mutual electrostatic repulsion of the cationic groups of the positively charged chains preserves an extended molecular conformation. This will lead to significant enhancement of the hydrodynamic radius of CPAA polymer chains in water, resulting in high dynamic viscosity. As the NaCl concentration increases in the polymer solution, the presence of NaCl facilitates charge screening and compresses the electrical double layer that diminishes these repulsive forces. This molecular interaction causes the polymer chains to coil, lowering the hydrodynamic radius and reducing chain entanglement [29,30,31]. Table 3, Table 4 and Table 5 quantify this behavior, reflecting the viscosity reduction % with NaCl addition.

### 3.5. Effect of CaCl_2_ Addition

**Figure 6:**
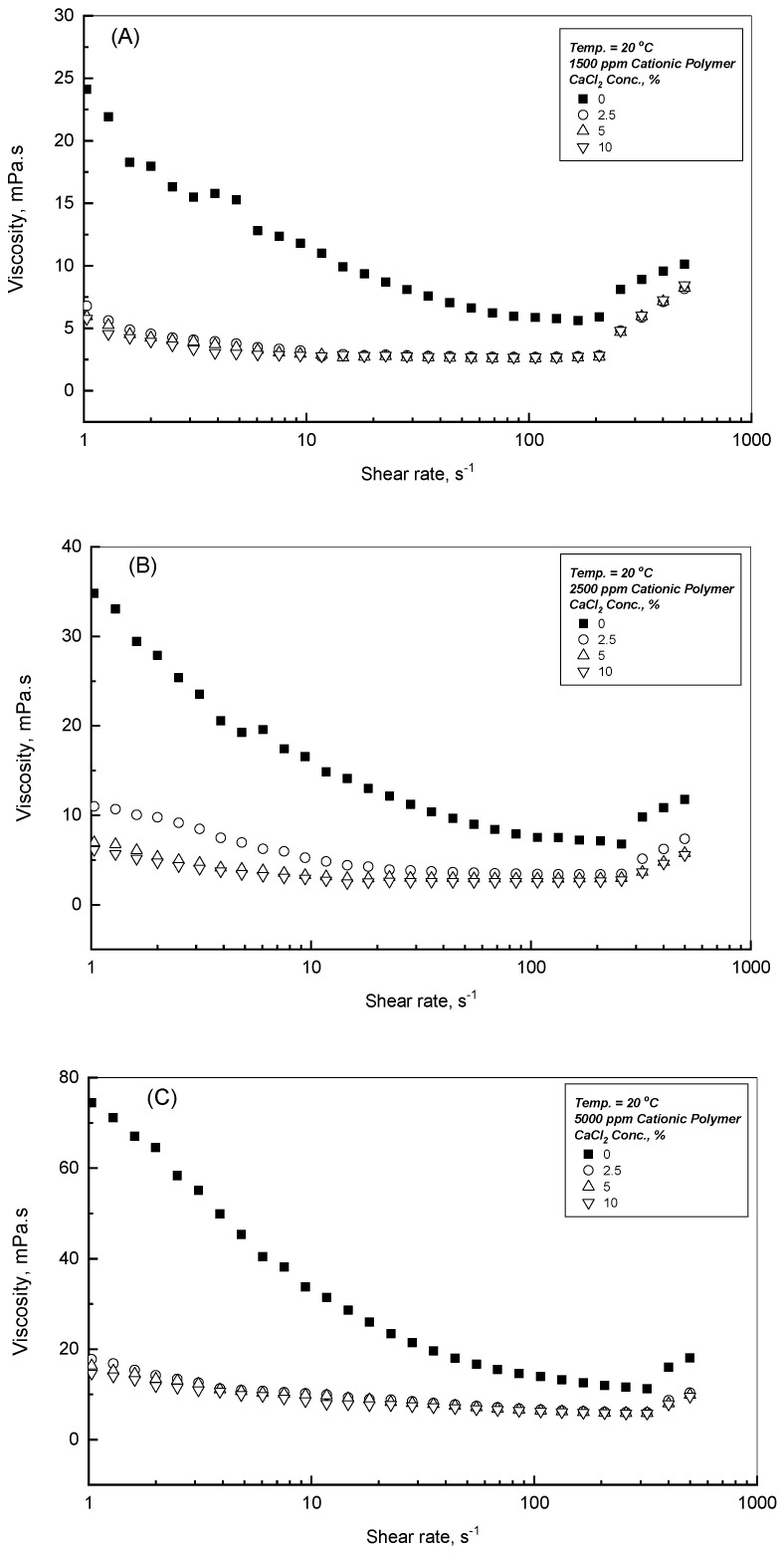
Effect of CaCl_2_ on the viscosity profiles for various CPAA concentrations at 20 °C. (**A**) 1500 ppm Cationic Polymer; (**B**) 2500 ppm Cationic Polymer; (**C**) 5000 ppm Cationic Polymer.

**Figure 7:**
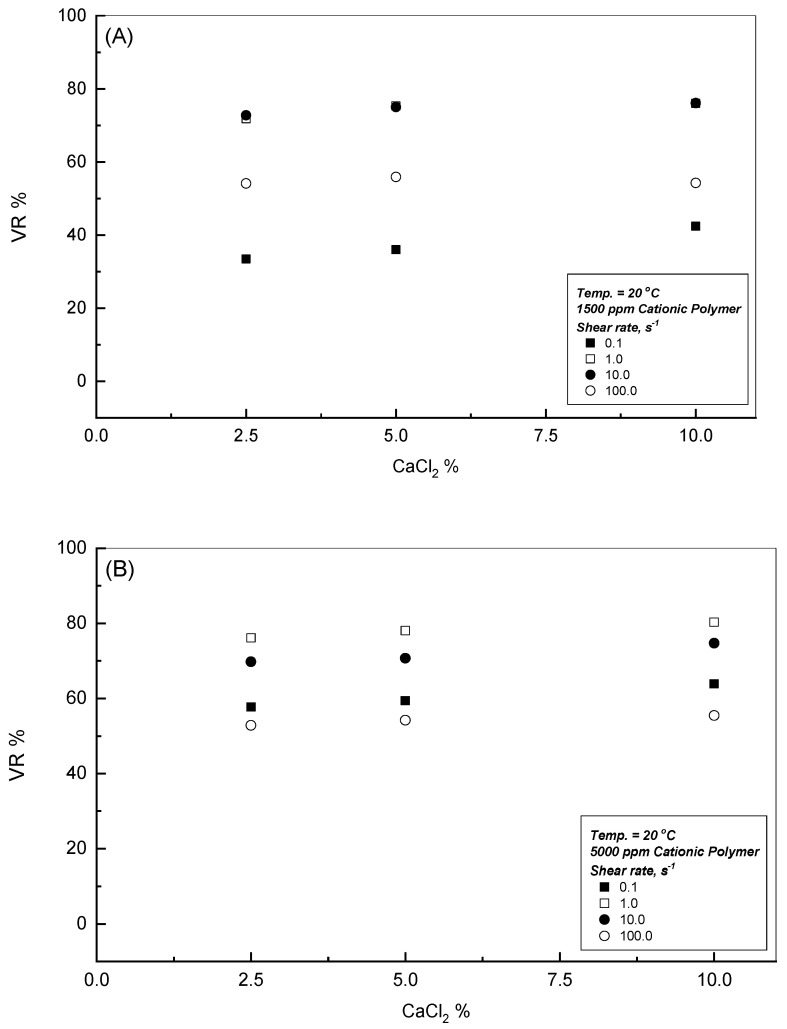
Effect of CaCl_2_ concentration on the viscosity reduction %. (**A**) 1500 ppm Cationic Polymer; (**B**) 5000 ppm Cationic Polymer.

To study the influence of CaCl_2_ presence on the flow behavior of CPAA solutions with different concentrations, Figure 6A–C display the viscosity profiles versus the applied shear rates at 20 °C for 1500, 2500, and 5000 ppm, respectively. This investigation inspects the effect of three CaCl_2_ concentrations of 2.5, 5, and 10 Wt.%, as indicated in the three associated figures. Owing to the repulsive forces that developed from the positive charges of the CPAA molecular chains within the double-distilled water, this leads to a higher hydrodynamic radius and consequently higher level of the measured dynamic viscosity, especially for higher concentrations of CPAA solutions [30,31]. Figure 6A–C display the influence of different additions of CaCl_2_ concentrations. For the initial CaCl_2_ addition of 2.5 Wt.%, the measured viscosity profile decreases, as per the results of the charge screening effect. This outcome is developed between the positive charges of the CPAA polymer chains and the negative cooperativity charges generated from the presence of CaCl_2_ within the solution, which reduces the hydrodynamic radius of the polymer molecular chains. However, beyond the presence of the 2.5 Wt.% CaCl_2_, Figure 6A–C show that no further significant viscosity reduction was reported. This remark can be observed clearly from Figure 7 for different CPAA concentrations.

**Figure 8:**
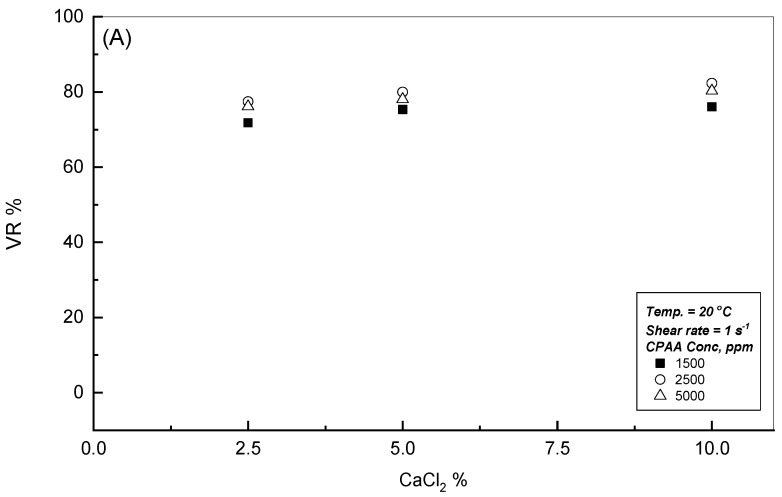

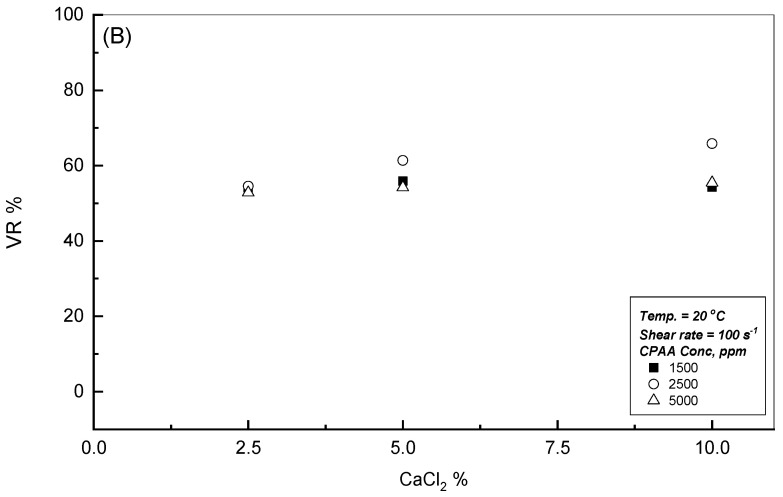
Effect of CaCl_2_% on the viscosity reduction % for different CPAA solutions. (**A**) Shear rate = 1 s^−1^; (**B**) Shear rate = 100 s^−1^.

A low concentration of 2.5 wt.% CaCl_2_ significantly reduces the viscosity of all CPAA solutions due to the high charge screening efficiency of divalent calcium ions. Beyond this 2.5 wt.% limit, no further substantial viscosity reduction was observed, as can be seen in Figure 6, Figure 7 and Figure 8. At low concentrations, calcium ions effectively shield the electrostatic repulsions between the cationic polymer chains, causing the hydrodynamic radius to contract and lowering the dynamic viscosity. At concentrations above 2.5 wt.%, the viscosity stabilizes as a result of a net balance between charge screening and the increased hydrophobicity of the polymer–solvent system. This stabilization suggests that once a critical ionic strength is reached, the CPAA molecular structure becomes resistant to further coiling, providing a stable viscosity profile suitable for high-salinity industrial applications [30,31].

**Figure 9:**
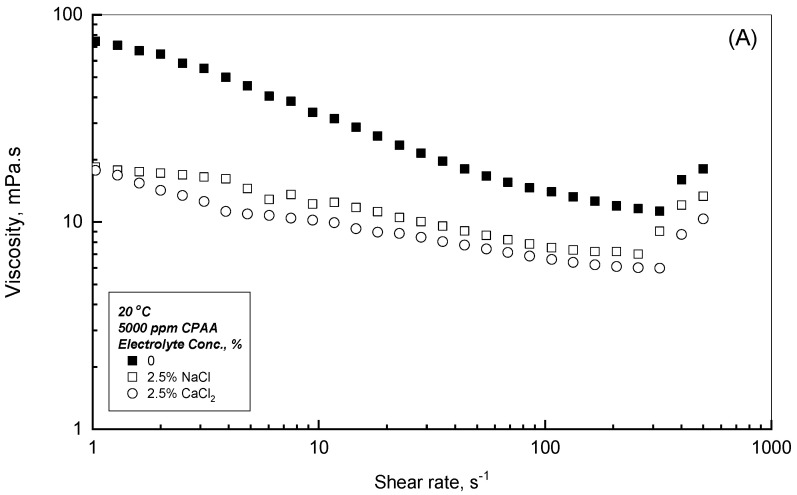

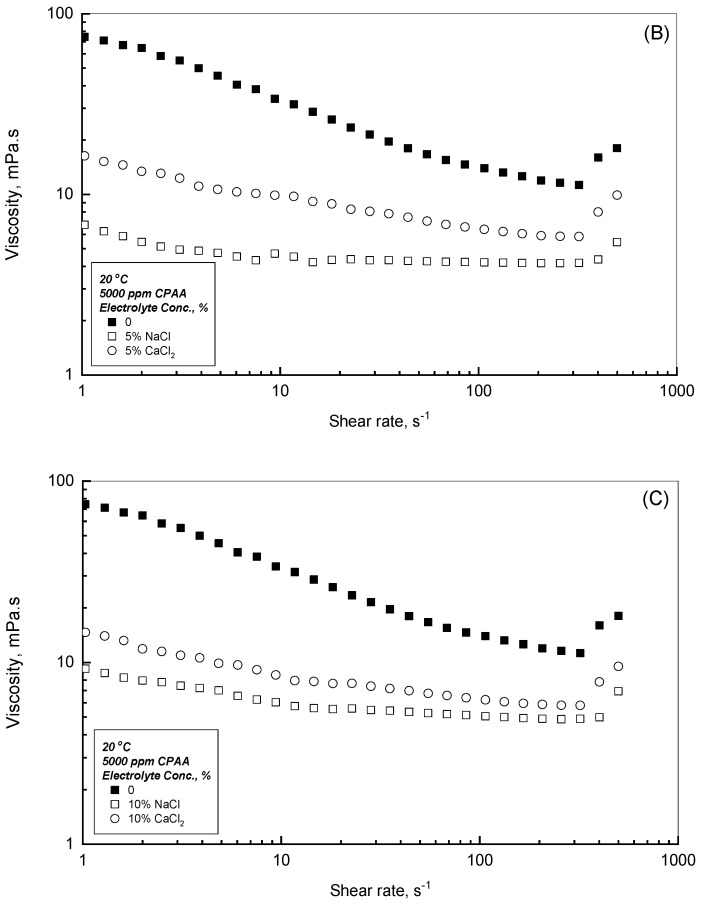
Effect of different electrolytes on the viscosity profile for 5000 ppm CPAA solutions. (**A**) Electrolyte Conc. = 2.5 Wt.%; (**B**) Electrolyte Conc. = 5 Wt.%; (**C**) Electrolyte Conc. = 10 Wt.%.

At this stage, it is essential to compare the effects of NaCl and CaCl_2_ on the flow behavior of CPAA solutions. Figure 9A–C illustrate the viscosity profiles of a 5000 ppm CPAA solution in double-distilled water as a representative example. Figure 9A shows that at a concentration of 2.5 wt.%, the divalent CaCl_2_ induces more significant viscosity reduction than the monovalent NaCl. This is attributed to the superior charge-screening capacity of divalent cations, which more effectively shrinks the polymer chain size and shields repulsive interactions between the polymer chains. Conversely, Figure 9B,C demonstrate that at higher electrolyte concentrations, CaCl_2_ does not produce additional viscosity reduction. This plateau results from the balance between electrostatic repulsion which lowers viscosity, and the increased solution hydrophobicity which enhances it, leading to insignificant viscosity changes beyond 2.5 wt.% CaCl_2_. 

Aqueous solutions of cationic polyacrylamide display complex rheological and morphological behaviors motivated by their positive charge density and high molecular weight, both of which are critical for industrial applications such as enhanced oil recovery and water treatment processes. As discussed earlier, these solutions exhibit non-Newtonian behavior of a strong shear-thinning profile, where dynamic viscosity decreases as the shear rate increases until the critical shear rate level. Furthermore, environmental factors such as elevated temperature and high salinity can cause a substantial reduction in solution viscosity.

From the morphological perspective of the cationic polyacrylamide solutions, the CPAA molecules generate intramolecular repulsion, forcing the polymer chains to uncoil into an extended conformation. This expansion increases their hydrodynamic volume and, consequently, the overall viscosity. The high charge density of CPAA further enhances this effect, creating a more rigid and expanded configuration due to intensified repulsion between chain segments. At higher concentrations, these long-chain molecules entangle to form a strong three-dimensional network [32,33].

## 4. Conclusions

The present detailed work evaluated the flow characteristics of cationic polyacrylamide polymer solutions. The following conclusions can be made:

1. The shear stress measurements increase with both shear rate and polymer concentration, up to a shear rate of 200 s^−1^. Beyond this level, the shear stress–shear rate profiles approach each other regardless the polymer concentration.
2. The obtained rheogram of CPAA declines significantly with the assigned temperature from 20 to 80 °C due to the thermal negative effect on the molecular interactions of the examined polymer solutions.
3. The CPAA polymer solutions with different concentrations display strong shear-thinning non-Newtonian flow profiles with shear rate in which the viscosity decreases considerably with shear rate until 200 s^−1^. Shear-thickening flow profiles will result with a shear rate higher than 200 s^−1^.
4. The results show significant increases in dynamic viscosity with CPAA concentration from 500 to 5000 ppm.
5. The measurements of the CPAA dynamic viscosity show strong deterioration with temperature over the range of 20 to 80 °C.
6. The existence of NaCl within the CPAA solutions will reduce the viscosity flow profiles significantly for all polymer concentrations, especially for the higher concentration of 5000 ppm.
7. The presence of 2.5 Wt.% CaCl_2_ in the CPAA solutions decreases the measurements of the dynamic viscosity values, while this effect is weakened with more addition of CaCl_2_ into the polymer solution.
8. For the addition of 2.5 Wt.% electrolyte, the presence of CaCl_2_ causes slightly higher viscosity reduction than in the case of NaCl. However, with the addition of higher electrolyte concentrations, the opposite behavior was reported, in which NaCl causes greater viscosity reduction than CaCl_2_.

This study describes the viscosity behaviors of CPAA solutions for pipeline flow and pump projects in industrial applications such as paper manufacturing (i.e., retention and drainage aids), oil and gas industries (i.e., enhanced oil recovery, drilling, and fracturing), water and wastewater treatments (i.e., sludge dewatering, flocculation, and clarification), and other industrial uses (i.e., textiles and dyeing, agriculture, building materials, and food processing). The described results are beneficial for high-temperature operations, such as improving the volumetric sweep of steam injection in EOR projects. Furthermore, identifying the critical shear rate for the commencement of shear-thickening is needed to avoid operational failure for pump cavitation and high-stress fluid transport operations. For further development, these outcomes permit the accurate preparation of CPAA solutions for high-viscosity operations including drilling muds and viscous fracturing fluids. The obtained results of the temperature effect on the viscosity profiles permit utilizing CPAA solutions in high-temperature reservoirs, confirming consistent consequences. In addition, this investigation enables the preparation of CPAA solutions for reservoir polymer injection in the presence of salinities, thus optimizing oil production while decreasing the costs accompanying high polymer concentrations.

## Figures and Tables

**Table 3:** Viscosity reduction for the 1500 ppm CPAA.

Shear Rate, s^−1^	2.5 Wt% NaCl	5 Wt% NaCl	10 Wt% NaCl
0.1	51.81	58.53	63.72
1.0	66.92	71.44	75.25
10.0	72.12	74.00	75.76

**Table 4:** Viscosity reduction for the 2500 ppm CPAA.

Shear Rate, s^−1^	2.5 Wt% NaCl	5 Wt% NaCl	10 Wt% NaCl
0.1	46.11	55.16	66.10
1.0	72.76	77.44	79.50
10.0	66.04	71.37	72.09

**Table 5:** Viscosity reduction for the 5000 ppm CPAA.

Shear Rate, s^−1^	2.5 Wt% NaCl	5 Wt% NaCl	10 Wt% NaCl
0.1	68.25	77.66	77.03
1.0	75.29	90.87	87.59
10.0	63.85	86.07	82.15

**Table 1:** Results of the Herschel–Bulkley model at 20 °C and 80 °C.

**Concentration, ppm**	**τ_o_, Pa**	**m, Pa.s^n^**	**n**	**R^2^**
500	0.0133	0.0002	1.4595	0.998
1500	0.0566	0.0007	1.4349	0.995
2500	0.1006	0.0005	1.4924	0.989
5000	0.2208	0.0011	1.4439	0.977
**Concentration, ppm**	**τ_o_, Pa**	**m, Pa.s^n^**	**n**	**R^2^**
500	0.0125	0.0001	1.4774	0.994
1500	0.0217	0.0002	1.4851	0.996
2500	0.0438	0.0018	1.1788	0.995
5000	0.0607	0.0084	0.9382	0.985

**Table 2:** Simulation analysis for the 5000 ppm CPAA solution at different temperatures.

Temperature, °C	τ_o_, Pa	m, Pa.s^n^	n	R^2^
20	0.2208	0.0011	1.4439	0.977
40	0.1429	0.0034	1.1747	0.977
80	0.0607	0.0084	0.9382	0.985

## Data Availability

All research data used were reported and displayed in the current manuscript.
